# Evaluation of marine zooplankton community structure through environmental DNA metabarcoding

**DOI:** 10.1002/lom3.10237

**Published:** 2018-01-17

**Authors:** Anni Djurhuus, Kathleen Pitz, Natalie A. Sawaya, Jaimie Rojas‐Márquez, Brianna Michaud, Enrique Montes, Frank Muller‐Karger, Mya Breitbart

**Affiliations:** ^1^ College of Marine Science, University of South Florida St Petersburg Florida; ^2^ Monterey Bay Aquatic Research Institute, Monterey California; ^3^ Fundación La Salle de Ciencias Naturales, Estacion de Investigaciones Marinas Isla de Margarita Venezuela

## Abstract

Zooplankton dominate the abundance and biomass of multicellular animals in pelagic marine environments; however, traditional methods to characterize zooplankton communities are invasive and laborious. This study compares zooplankton taxonomic composition revealed through metabarcoding of the cytochrome oxidase I (COI) and 18S rRNA genes to traditional morphological identification by microscopy. Triplicates of three different sample types were collected from three coral reef sites in the Florida Keys National Marine Sanctuary: (1) 1 L surface seawater samples prefiltered through 3 *μ*m filters and subsequently collected on 0.22 *μ*m filters for eDNA (PF‐eDNA); (2) 1 L surface seawater samples filtered on 0.22 *μ*m pore‐size filters (environmental DNA; eDNA), and (3) zooplankton tissue samples from 64 *μ*m, 200 *μ*m, and 500 *μ*m mesh size net tows. The zooplankton tissue samples were split, with half identified morphologically and tissue DNA (T‐DNA) extracted from the other half. The COI and 18S rRNA gene metabarcoding of PF‐eDNA, eDNA, and T‐DNA samples was performed using Illumina MiSeq. Of the families detected with COI and 18S rRNA gene metabarcoding, 40% and 32%, respectively, were also identified through morphological assessments. Significant differences in taxonomic composition were observed between PF‐DNA, eDNA, and T‐DNA with both genetic markers. PF‐eDNA resulted in detection of fewer taxa than the other two sample types; thus, prefiltering is not recommended. All dominant copepod taxa (> 5% of total abundance) were detected with eDNA, T‐DNA, and morphological assessments, demonstrating that eDNA metabarcoding is a promising technique for future biodiversity assessments of pelagic zooplankton in marine systems.

An essential element of environmental conservation and monitoring programs is biodiversity assessment, including describing community taxonomic composition at different trophic levels (Lodge et al. 2012). Traditional methods that characterize biodiversity are laborious (i.e., visual surveys) and can be environmentally destructive (e.g., trawling) (Wheeler et al. 2004; Wheeler and Valdecasas 2005). Genetic analysis of environmental DNA (eDNA), which contains DNA shed by organisms present in a given environment, offers a high‐throughput, cheaper, more sensitive, and less destructive method for characterizing biodiversity (Davy et al. 2015; Flynn et al. 2015; Harvey et al. 2017). The estimation of biodiversity from metabarcoding (PCR and next‐generation sequencing) of conserved genetic markers has become standard practice in the field of microbial ecology (Rusch et al. 2007; Caporaso et al. 2012). For multicellular organisms, the application of specific genetic assays to eDNA is frequently used for the detection of rare or invasive organisms (Ardura et al. 2015). Metabarcoding of eDNA is becoming increasingly applied for determining taxonomic composition of higher trophic levels in aquatic and terrestrial environments (Aylagas et al. 2016; Kelly et al. 2016; Port et al. 2016; Valentini et al. 2016; Kelly et al. 2017). Such studies complement traditional surveys and frequently lead to the identification of organisms not commonly detected using visual techniques (Kelly et al. 2016; Olds et al. 2016). The challenge has been to relate this increased species detection to traditional methods of biodiversity assessment.

Metabarcoding of eDNA has not been thoroughly evaluated for assessing the biodiversity of marine zooplankton communities. Zooplankton dominate the abundance and biomass of multicellular pelagic animals (Schminke 2007). Holoplankton (e.g., copepods, chaetognaths) and meroplankton (e.g., fish larvae, crab larvae) communities are highly diverse, occupy a variety of niches, and contribute to ecosystem functions (Richardson 2008; Steinberg et al. 2008). Zooplankton play important roles in biogeochemical cycling through the biological pump and by transferring energy to higher trophic levels (Ward et al. 2012; Turner 2015). Despite their ecological importance, the spatiotemporal variability in the composition of zooplankton assemblages is not well characterized, primarily due to challenges with taxonomic identification. Many samples collected during oceanographic expeditions or monitoring surveys for zooplankton taxonomic composition studies are examined only partially, or not at all (Roger et al. 2000; Schminke 2007).

Life in the sea is changing (Butchart et al. 2010) and techniques for analyzing the biodiversity of communities across trophic levels are required to advance ecological research and ecosystem‐based management. Documenting such methods to facilitate wider use is one of the goals of the Marine Biodiversity Observation Network (MBON) a subdivision of the Group on Earth Observations Biodiversity Observation Network (GEO BON; Muller‐Karger et al. 2014). eDNA metabarcoding offers a practical means for assessing biodiversity over time, from tropical to polar ecosystems, and at multiple trophic levels, to inform policy and management across local, regional, national, and international scales.

To achieve this MBON vision, we tested the effectiveness of eDNA metabarcoding for determining zooplankton taxonomic composition and monitoring community responses to environmental change. Previous studies have performed metabarcoding of cytochrome oxidase I (COI) and 18S rRNA genes on zooplankton community tissues (T‐DNA; i.e., net tow biomass), revealing moderately accurate detection levels (i.e., correlations) between biomass and the relative amount of sequences recovered for particular taxa (Lindeque et al. 2013; Harvey et al. 2017). However, noninvasive methods for assessing biodiversity, particularly metabarcoding of eDNA collected from surface water samples, have not previously been tested for pelagic zooplankton communities.

Here, we assess the taxonomic composition of the zooplankton community at three coral reef sites within the Florida Keys National Marine Sanctuary using metabarcoding of eDNA and T‐DNA with two genetic loci (COI and 18S) compared to traditional microscopy surveys (morphological identification). This study is the first to our knowledge to compare eDNA metabarcoding data for pelagic marine zooplankton taxa with morphological taxonomic data for net tows. Based on previous studies (Lindeque et al. 2013; Harvey et al. 2017), we hypothesized that the patterns of morphological zooplankton taxonomic composition from net tows would be most similar to results obtained with T‐DNA metabarcoding. Additionally, we tested the impact of applying a prefiltering step to the collection of eDNA, to reduce biases associated with capturing whole animals. This work lays the foundation for applying eDNA metabarcoding to marine pelagic zooplankton communities and provides insight for comparing results obtained using this method to traditional techniques.

## Methods

### Sample collection

Sampling was carried out on the R/V Walton Smith (University of Miami) as part of the South Florida Program (NOAA Atlantic Oceanographic and Meteorological Laboratory/AOML). Samples were collected at three stations in March 2016: Molasses Reef (25.05163° N, −80.2285° W; 15^th^ March), Looe Key (24.32302° N, −81.24806° W; 16^th^ March), and Western Sambo (24.28604° N, −81.42889° W; 16^th^ March). At each station, triplicate horizontal hauls (surface water) were performed with 64 *μ*m, 200 *μ*m, and 500 *μ*m mesh size Bongo plankton nets. Each tow lasted 5 min at 1 knot. We allowed at least 15 min between replicate net tows to sample an undisturbed water column (Jacobs and Grant 1978).

**Figure 1:**
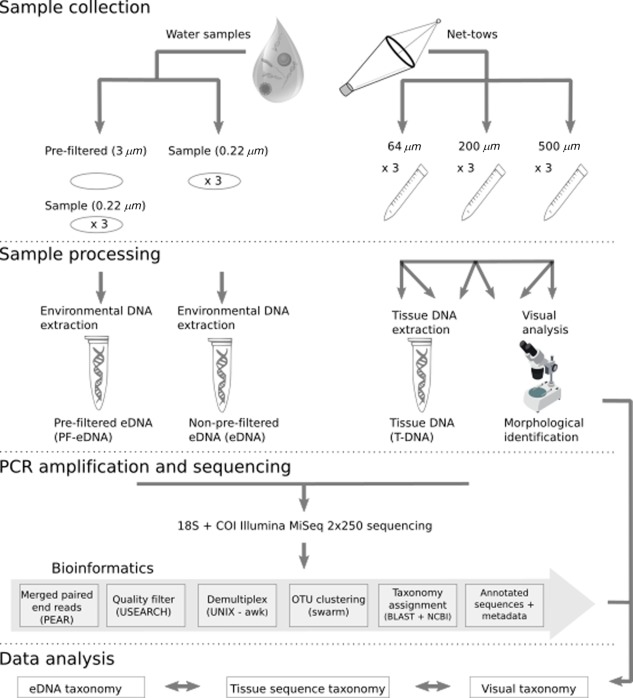
Schematic of sample collection and processing pipeline. One‐liter water samples were collected and filtered for environmental DNA (eDNA) genetic analyses (left) and tissue samples were collected for DNA and morphological analyses (right). Prefiltered eDNA (PF‐eDNA), eDNA, and tissue DNA (T‐DNA) samples were amplified using primers for the 18S rRNA (Amaral‐Zettler et al. 2009) and COI (Folmer et al. 1994; Leray et al. 2013) genes and sequenced on an Illumina MiSeq platform.

The 64 *μ*m, 200 *μ*m, and 500 *μ*m zooplankton samples were halved using a Folsom plankton splitter. One fraction was preserved in 15% formalin for morphological analysis. The other half was frozen immediately at −20°C for genetic zooplankton community analysis (“T‐DNA”; Fig. 1).

For eDNA analyses, triplicate 1 L surface water samples (∼ 0.5 m depth) were collected from each station using Niskin bottles arranged on a rosette. The samples were filtered onto 0.22 *μ*m PVDF Sterivex filters (Millipore, U.S.A.). To test the effect of prefiltering the eDNA sample (PF‐eDNA), another set of triplicate 1 L surface water samples was collected from each station. These were prefiltered through a 3 *μ*m pore‐size flat nitrocellulose filter (Millipore, U.S.A.) and then filtered on to a 0.22 *μ*m PVDF Sterivex filter (Millipore, U.S.A.) (Fig. 1). All filters (both eDNA and PF‐eDNA) were flash‐frozen in liquid nitrogen, and preserved at −80°C.

Zooplankton identification methods will henceforth be referred to as “PF‐eDNA” for prefiltered environmental DNA metabarcoding, “eDNA” for non‐prefiltered environmental DNA metabarcoding, “T‐DNA” for total zooplankton net tow tissue metabarcoding, and “morphological” for microscope‐based visual analysis (Fig. 1). We collected a total of nine samples each (three from each station) for PF‐eDNA and eDNA, in addition to nine samples per station for T‐DNA and morphological analyses with triplicates of each net tow size fraction (Fig. 1).

### Metabarcoding

#### DNA extraction

DNA was extracted from the PF‐eDNA, eDNA, and T‐DNA using the DNeasy Blood and Tissue Kit (Qiagen, U.S.A.). Two extraction blanks (i.e., an extraction with no filter) and two filtration blanks (1 L of MilliQ water filtered through a Sterivex filter, then processed alongside the samples) were included as controls.

Each T‐DNA sample was thawed, pelleted by centrifugation (3000 x g for 5 min.), and the supernatant was removed with a sterile pipette. The pelleted zooplankton biomass was then homogenized using a 10 mL syringe and a 19 G needle. According to Lindeque et al. (2013). The DNA extraction protocol was modified to include an initial bead‐beating step. Specifically, 1 g of 0.5 mm and 1 g of 0.1 mm glass beads (BioSpec Products) along with 900 μL ATL Buffer (Qiagen) were added to each tube. Before use, the glass beads were sterilized by combustion at 500°C for 3 h. Tubes were shaken on a vortexer with a bead‐beater adapter at maximum speed for 45 s, followed by incubation at 56°C for 30 min and a second round of bead beating and incubation. Next, 100 μL of Proteinase K (2 mg/L final concentration) was added to each tube, vortexed for 10 s, and incubated at 56°C for 2 h with shaking. Samples were vortexed for 15 s and centrifuged for 1 min at 4000 × *g*. The supernatant (∼ 900 μL) was then transferred to a new 2‐mL tube and centrifuged for 1 min at 13,000 × *g*. Then, 650 μL of bead‐free supernatant was transferred to a new 2‐mL tube. Thereafter, the manufacturer's protocol was followed with the following modifications: 650 μL AL Buffer, 650 μL ethanol, and final elution steps of 2 × 50 μL AE Buffer for each sample.

#### PCR and library preparation

DNA extracts were amplified with primer sets targeting the 18S rRNA gene, sequences as follows (5′‐3′): 1391F, GTACACACCGCCCGTC, EukBr, TGATCCTTCTGCAGGTTCACCTAC (Amaral‐Zettler et al. 2009) and the COI gene, sequences as follows (5′‐3′): mlCOIintF, GGWACWGGWTGAACWGTWTAYCCYCC and HCO2198, TAAACTTCAGGGTGACCAAAAAATCA (Folmer et al. 1994; Leray et al. 2013). The PCR reaction mixture was the same for both genes. PCR was performed in triplicate 25 *μ*L reactions for each sample using 12‐basepair Golay barcoded reverse primers (Amaral‐Zettler et al. 2009). Each reaction was carried out using 1 *μ*L DNA extract at a 1 : 10 dilution, 10 *μ*L AmpliTaq Gold master mix (Thermo Fisher Scientific, U.S.A.), 1 *μ*L each of forward and reverse primers (5 *μ*M), 8 *μ*L molecular‐biology grade water (Sigma‐Aldrich, U.S.A.), and 4 *μ*L of 10 *μ*M mammalian blocking primer (GCCCGTCGCTACTACCGATTGG/ideoxyI//ideoxyI//ideoxyI//ideoxyI//ideoxyI/TTAGTGAGGCCCT/3SpC3/) for the 18S rRNA gene only (Earth Microbiome Project; Vestheim and Jarman 2008). PCR reactions were run in triplicate on 96‐well plates with a negative (no template added) control on each plate. 18S rRNA cycling parameters were 94°C for 3 min; 35 cycles at 94°C for 45 s; 65°C for 15 s; 57°C for 30 s; and 72°C for 90 s; COI cycling parameters were 95°C for 10 min; 16 cycles at 94°C for 10 s; 62°C for 30 s (decreasing by 1°C per cycle); 68°C for 60 s; 25 cycles at 94°C for 10 s; 46°C for 30 s; 68°C for 60 s; and 72°C for 10 min.

Triplicate PCR products were pooled and quality was confirmed by agarose gel electrophoresis (1.5%). PCR products were purified and size‐selected using the Agencourt AMPure XP bead system (Beckman Coulter, U.S.A.). A second agarose gel was run to confirm primer removal and retention of target amplicons after purification. Purified products were quantified with a Qubit dsDNA HS Assay Kit (Invitrogen, U.S.A.). Equimolar concentrations of 10 nM/sample were combined into a single library pool. All sequencing was performed at the Stanford Functional Genomics Facility on an Illumina MiSeq platform using paired‐end sequencing (MiSeq Reagent kit v2) and a 20% PhiX174 spike‐in control to improve the quality of low‐diversity samples (Kircher et al. 2009).

#### Bioinformatics

Sequence data were processed using a Unix shell script written to analyze Illumina‐generated eDNA metabarcoding data (https://github.com/jimmyodonnell/banzai). The following steps were executed with the pipeline: merging of paired reads using PEAR v0.9.2 (Zhang et al. 2014), quality filtering with USEARCH (Edgar 2010), and primer removal with cutadapt v.1.4.2 (Martin 2011) allowing for no mismatches in the primer sequence. Operational Taxonomic Unit (OTU) clustering was done using Swarm (cluster radius of 1) (Mahé et al. 2014), taxonomic annotation by nucleotide BLAST (BLASTN) (Altschul et al. 1990) against the NCBI nt reference database (e‐value: 1 × 10^−5^), and secondary taxonomic assignment using the lowest common ancestor (LCA) algorithm in MEGAN at 80% (Huson et al. 2007). For both primer sets, reads with homopolymers > 7 bases were also omitted. All data from this study can be accessed from GenBank accession no. PRJNA412886.

The OTU tables were filtered for contaminants (i.e., humans, cows, or dogs) using a suite of ad hoc R scripts developed by the U.S. MBON project (https://github.com/marinebon/MBON) (Djurhuus et al. 2017). We also removed all prokaryotes, protists, arachnids, hominids, fungi, and phytoplankton from the data analysis using the R package Phyloseq (based on taxonomic annotation) to focus exclusively on zooplankton for the purpose of this manuscript.

### Morphological taxonomy

Morphological identification was performed according to International Council for the Exploration of the Sea (ICES) protocols (Roger et al. 2000) aiming to count at least 200 animals per sample. Due to a high number of organisms, we subsampled to make counting feasible. For the 64 *μ*m and 200 *μ*m net tows, zooplankton were resuspended in 300 mL of sterile water and a 5–10 mL aliquot was removed, depending on the number of animals, using a Stempel pipette. Zooplankton were then identified and counted. After each initial pass, the entire sample was scanned for the presence of previously undetected genera or species.

Animals collected with 500 *μ*m net tows were identified by microscopy from either a full sample, a 1/4, or a 1/8 fraction of the sample. Subsampling was done with a Folsom splitter until approximately 200–300 animals were retained. Net tows were performed during copepod‐spawning periods; consequently, copepod nauplii were abundant. Since copepod nauplii are difficult to identify morphologically, we did not attempt to determine their taxonomy. Adult copepods were identified to genus or species level. Other zooplankton were identified to phylum and, where possible, to class, order, or family (i.e., Chaetognatha and Euphausiacea).

Zooplankton abundances (density; individuals m^−3^) were calculated by dividing animal counts by the product of net mouth area, tow speed, and tow duration (i.e., volume of water filtered by the net). Abundances of gastropods, the copepod order Harpacticoida, and the genera *Paracalanus* and *Oithona* were converted to biomass after Kelble et al. (2010). These group‐specific biomass estimates were compared to sequence abundances from the T‐DNA samples from each station across all size fractions, to evaluate possible correlations.

### Data analysis

The OTU table was randomly subsampled to a depth of 9484 and 9563 sequences per sample for 18S rRNA and COI genes, respectively. Rarefaction accounts for uneven sampling depth obtained via high‐throughput sequencing (McMurdie and Holmes 2014). All statistical analyses were performed using the R package vegan (Oksanen et al. 2013).

Analyses of variance (ANOVA) followed by Tukey Honest Significant Difference (Tukey HSD) tests were performed to determine whether species richness differed significantly across methods. Nonmetric multidimensional scaling (NMDS) was performed using the Bray‐Curtis dissimilarity indices on the triplicate sequencing analyses (PF‐eDNA, eDNA, and T‐DNA), with the *metaMDS* function from R package Phyloseq (McMurdie and Holmes 2012), at the OTU level. We used the Adonis function (vegan) (Oksanen et al. 2013) to parse the data according to different treatments (PF‐eDNA, eDNA, and T‐DNA). A Permutation Analysis of Variance (PERMANOVA) was done to address significance in taxonomic composition differences at the OTU level.

Triplicate sequencing data for each sample were averaged with Phyloseq (McMurdie and Holmes 2013). To compare morphological and sequencing data, we grouped taxonomic results at the Class level. We performed a detailed analysis on the holoplankton, specifically the infraclass Neocopepoda (Maxillopoda), containing the orders Harpacticoida, Calanoida, and Poecilostomatoida. All data were plotted with Phyloseq, superheat (Barter and Yu 2015), and ggplot2 (Wickham 2009). All statistical analyses were done using the R software package (R Development Team 2009).

## Results

### Molecular taxonomy

#### Observed richness

**Figure 2:**
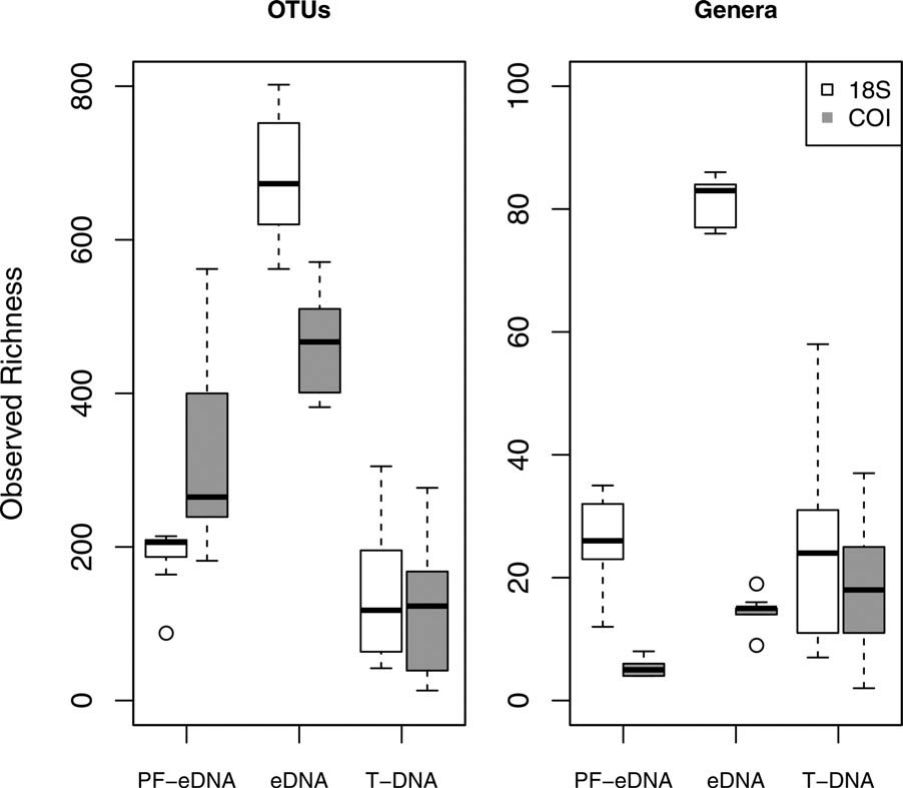
Boxplot of observed richness on an OTU level (left) and for OTUs assigned to genus (right) for the two genetic loci (18S rRNA and COI) from prefiltered environmental DNA (PF‐eDNA), environmental DNA (eDNA), and tissue DNA (T‐DNA).

DNA metabarcoding resulted in the identification of 12,639 OTUs from the 18S rRNA gene, and 9907 OTUs from the COI gene. Overall, observed OTU richness was similar between the two genetic markers (18S rRNA and COI) and the individual treatments (PF‐eDNA, eDNA, and T‐DNA). One exception to this was the increased richness observed for the eDNA 18S rRNA gene data (Tukey HSD, *p* < 0.01, Fig. 2). The lowest OTU richness was observed for T‐DNA using both genetic loci (Tukey HSD, *p* < 0.01, Fig. 2). Approximately, 25% and 50% of the sequences were taxonomically assigned to the genus level for 18S rRNA and COI genes, respectively (Table 1). The eDNA COI data yielded a much lower richness when restricted to sequences annotated to the genus level. At the genus level, eDNA 18S rRNA sequences showed a significantly higher richness than T‐DNA and PF‐eDNA (Tukey HSD, *p* < 0.05). PF‐eDNA 18S rRNA data had richness similar to T‐DNA for both genetic markers. However, prefiltering decreased the recovered richness compared to the non‐prefiltered eDNA.

**Table 1:** Results of metabarcoding of 18S rRNA and COI loci from all combined sequences, compared to morphologically assigned taxonomy. Not all groups were identified to family with morphological taxonomy, in which case they were identified to order or phylum (e.g., Chaetognatha were assigned at phylum but not family level). If not assigned to a family, the organism was added to the total unique families on the order or phylum level.

	18S	COI	Morpho	Total
Total reads	8,649,990	2,979,556	—	11,629,546
Total OTUs	12,639	9907	—	22,546
Annotated reads	2,073,960	1,126,844	—	3,200,804
Annotated OTUs	1952	952	—	2904
Families or groups	409	122	55	—

#### Community differences

**Figure 3:**
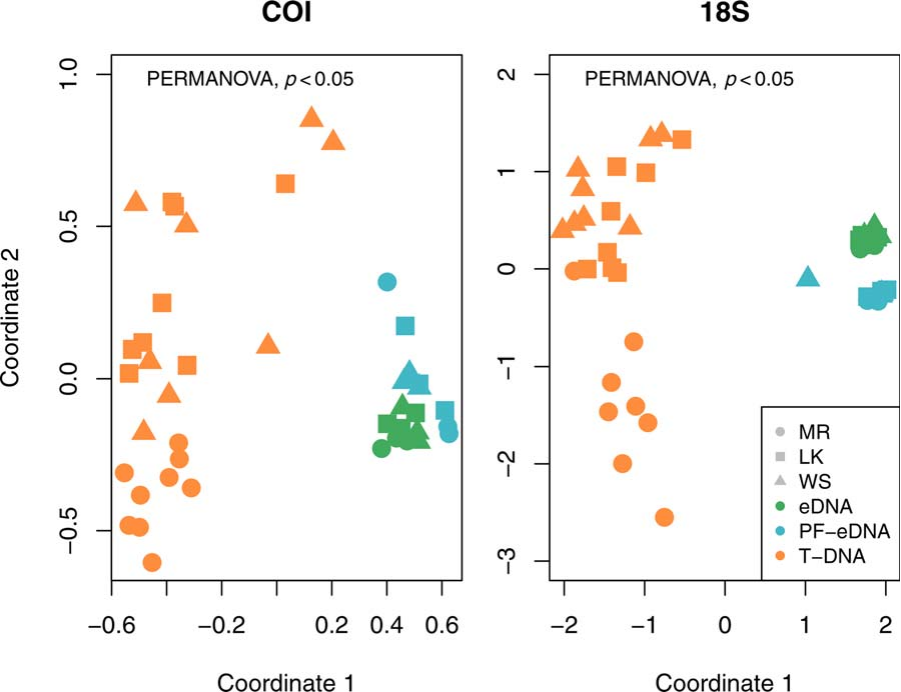
Nonmetric multidimensional scaling plot (NMDS) of all sequenced samples at the OTU level. The three sampling stations are Molasses Reef (MR), Looe Key (LK), and Western Sambo (WS). There was a statistically significant difference among the prefiltered environmental DNA (PF‐eDNA), environmental DNA (eDNA), and tissue DNA (T‐DNA) (*p* < 0.05). The PF‐eDNA and eDNA samples were more similar to each other compared to the T‐DNA. The T‐DNA had a higher spread in variance among sites. However, triplicate samples of T‐DNA (from locations and mesh size) were not significantly different from each other (*p* > 0.05).

**Figure 4:**
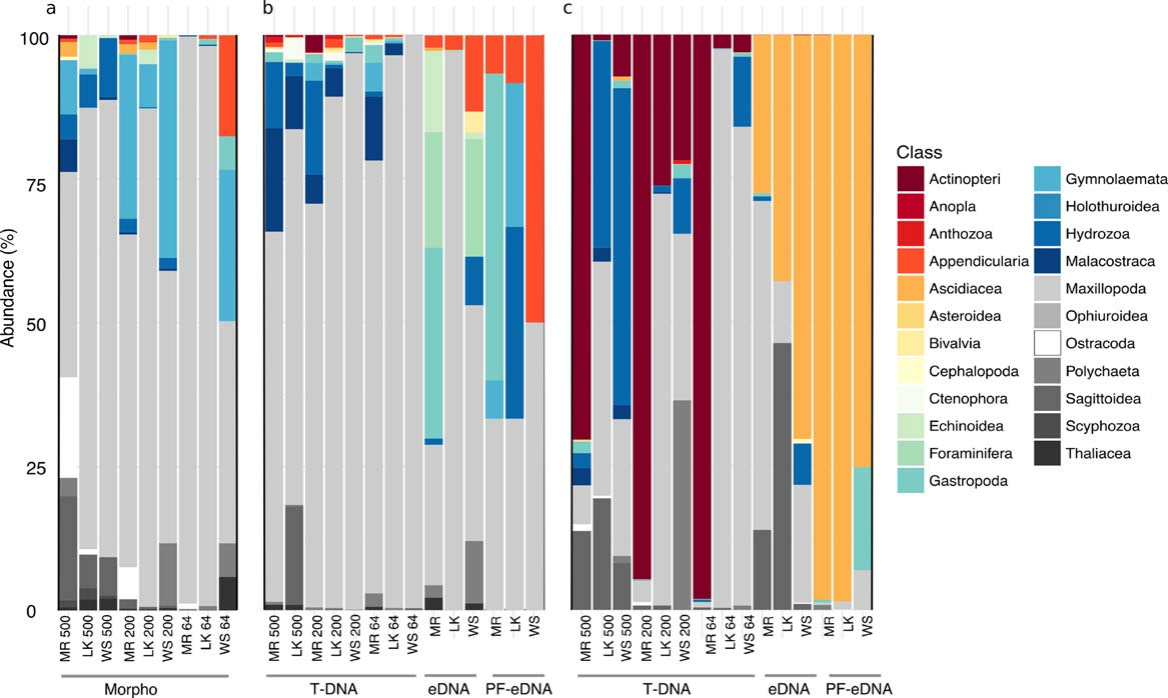
Barplot of all data at the Class level. (**a**) Morphology, (**b**) 18S rRNA, (**c**) COI. The two genetic loci (COI and 18S rRNA) recover different organisms on a Class level (i.e., chordates: ray‐finned fishes [Class Actinopteri] and ascidians [Class Ascidiacea]). The tissue DNA (T‐DNA) resembled the zooplankton taxonomic composition identified by microscopy more closely than the environmental DNA (eDNA) or prefiltered environmental DNA (PF‐eDNA). The morphologically identified taxa were more similar to the 18S rRNA taxonomy than to the COI. Both genes detected the same taxonomic groups but at quite different abundances. The numbers on the labels for each bar refer to the mesh size (in *μ*m) used in the net tows, and letters represent the sampling stations (Molasses Reef [MR], Looe Key [LK], and Western Sambo [WS]).

**Figure 5:**
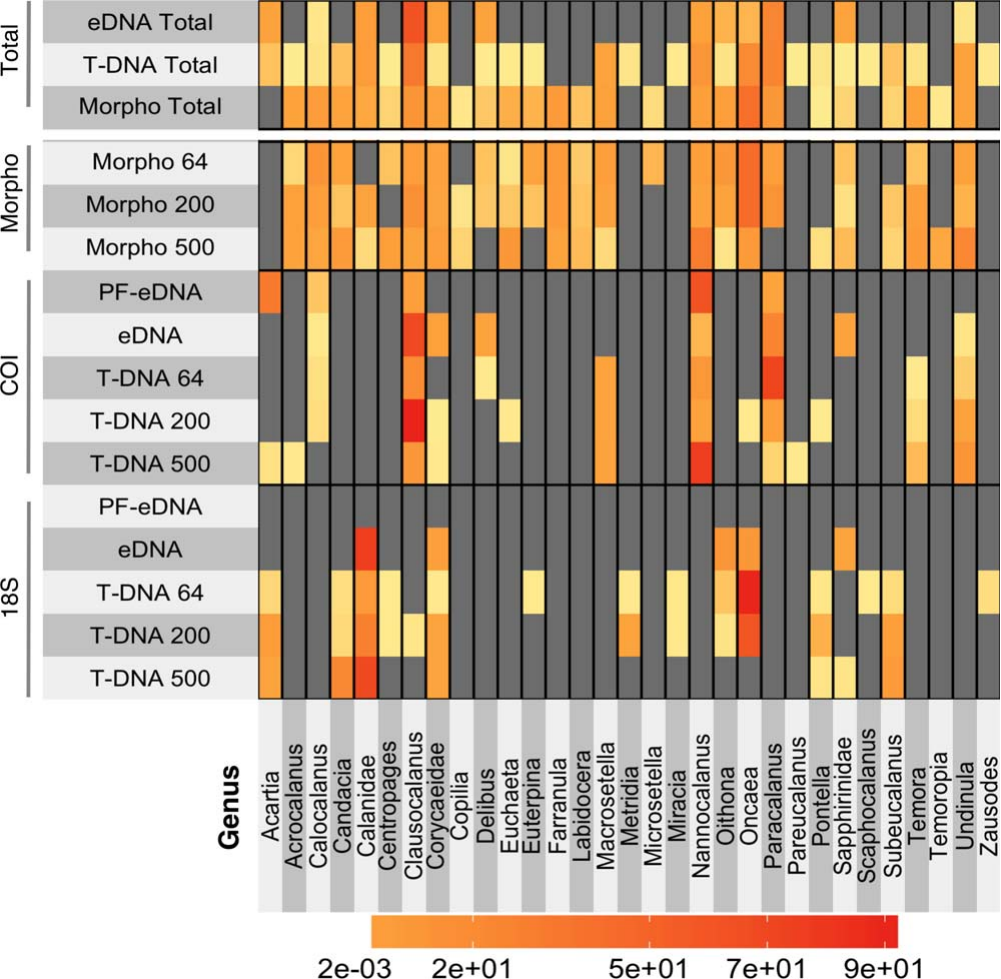
Heatmap of all copepod data compared between the two loci, 18S rRNA and COI, and morphological assessment. The totals at the top represent both genetic loci for PF‐eDNA and eDNA combined, T‐DNA of all three size fractions, and morphology of all three size fractions. The numbers on the left side refer to the mesh size used in the net tows. The color scale indicates relative abundance between the different samples.

**Figure 6:**
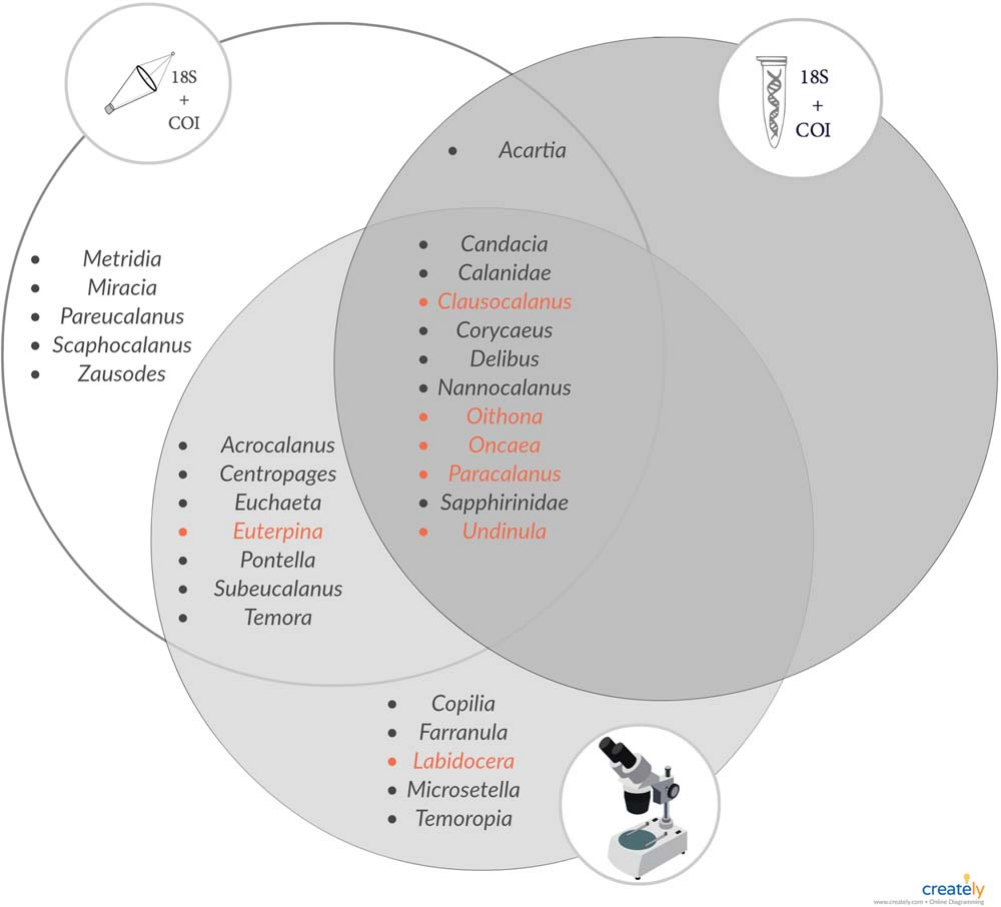
Venn diagram of copepod genera detected using genetic markers in tissue DNA (T‐DNA, left), environmental DNA (eDNA, right), and morphological assessments (bottom). Nearly all dominant (red, > 5% of total abundance) copepod genera were identified by each treatment type.

Based on nonmetric multidimensional scaling (NMDS) prior to pooling data from triplicates or sites, the PF‐eDNA, eDNA, and T‐DNA sample types each showed significantly different taxonomic composition at the OTU level (PERMANOVA, *p* < 0.05). The T‐DNA samples had a larger variance than the PF‐eDNA and eDNA samples, although there were significant differences among all three treatments for both markers (Fig. 3). The T‐DNA 18S rRNA sequences were dominated by Maxillopoda (including barnacles, copepods, and related arthropods), with relatively higher counts of Malacostraca (including shrimp and amphipods) at Molasses Reef (Fig. 4). The 18S rRNA sequences from the PF‐eDNA and eDNA samples were almost exclusively annotated to the classes Maxillopoda, Gastropoda, Malacostraca, Foraminifera, and Appendicularia. The PF‐eDNA samples contained significantly fewer species of metazoans (ANOVA *p* < 0.05) than the eDNA and T‐DNA for both 18S rRNA and COI (Figs. 4, 5, 6).

Large differences were detected among the stations using COI, especially for T‐DNA. Most T‐DNA samples from Looe Key and Western Sambo were dominated by a mixture of the classes Actinopteri (Phylum: Osteichtyes), Maxillopoda (Superclass: Arthropoda), Hydrozoa (Phylum: Cnidaria), and Sagittoidea (Phylum: Chaetognatha). All samples from Molasses Reef were dominated by ray‐finned fishes (Class: Actinopteri) (Fig. 4). From the three size fractions of net tows, the only samples from the COI T‐DNA samples dominated by Maxillopoda were Looe Key and Western Sambo at 64 *μ*m. For COI, the PF‐eDNA and eDNA samples were mostly dominated by ascidians (Class Ascidiacea), although the eDNA recovered additional classes.

### Morphological taxonomy

All samples collected for morphological assessments using microscopy were dominated by the class Maxillopoda (Arthropoda) (Fig. 4). This was similar to the 18S rRNA sequencing results. There were slight differences in relative abundance among the different stations and also among net tow mesh sizes (Fig. 4). Net tows with a 500 *μ*m mesh size recovered more of the larger animals, such as arrow worms (Family Sagittoidea, Phylum Chaetognatha) and jellyfishes (Family Scyphozoa, Phylum Cnidaria). The morphological samples collected at Western Sambo with the 64 *μ*m and 200 *μ*m mesh sizes and at Molasses Reef with the 200 *μ*m mesh size were the most different, due to high abundances of bryozoans (specifically, Class Gymnolaemata, Phylum Bryozoa).

### Comparison of molecular and morphological taxonomy

There was a large overlap between families detected using morphological vs. molecular techniques (COI: ∼ 40%, 18S rRNA: ∼ 32%) (Supporting Information Fig. S1). The families identified in eDNA that were not detected by microscopy were most frequently sessile animals such as corals and sponges that would not be captured with net tows. Overall, most sequences were annotated to Maxillopoda (COI: 31.6%, 18S rRNA: 66.35%). For the eDNA 18S rRNA data, a large fraction of sequences was annotated to Maxillopoda, Malacostraca, Appendicularia, and Gastropoda, and for COI‐eDNA relatively more were annotated as ascidians (Class Ascidiacea) regardless of site (Fig. 4). Most of the sequences that were annotated as chordates were ascidians (Class Ascidiacea; 37%) and ray‐finned fishes (Class Actinopteri; 52.2%). No chordate sequences were observed among the 18S rRNA sequences.

*Paracalanus* was not detected with the 18S rRNA gene, and *Oithona* was not detected with COI. Thus, they could not be compared to biomass using those markers. In some cases, there were co‐occurring elevated biomass and sequence abundances, although these relationships were never statistically significant (Supporting Information Fig. S2). Unfortunately, biomass conversion factors were only available for the copepod genera *Paracalanus* and *Oithona*, the copepod Order Harpacticoida, and Gastropoda (Kelble et al. 2010). Having conversion factors for more groups and a higher replication of sequenced samples could help conduct additional comparisons between biomass and sequence abundance to get a more meaningful correlation.

#### Maxillopoda (Copepoda)

Combined, morphological and genetic methods identified a total of 31 different genera of the subclass Copepoda from all samples (Figs. 5, 6). Eleven of these copepod genera, including most of the dominant taxa, were identified by all three methods (morphological and metabarcoding of COI and 18S rRNA from both eDNA and T‐DNA) (Fig. 6). Sixteen different copepod genera were detected using 18S rRNA gene sequencing, 16 genera using COI sequencing, and 25 genera using morphological assessment. Only 12 copepod genera were detected in eDNA (Figs. 5, 6). All copepod genera detected in eDNA were also found in T‐DNA.

The PF‐eDNA did not yield any sequences annotated to Maxillopoda from the 18S rRNA sequences and only 5 genera were detected with COI. Only 5 and 8 genera were detected from the 18S rRNA and COI sequence data, respectively, from the eDNA samples. Only two families, Corycaidea and Sapphirinidae (only assigned to family), overlapped between the two genetic markers. T‐DNA obtained using different net mesh sizes contained large differences in the detected genera. When sequences from both loci (18S rRNA gene and COI) from all size fractions of the T‐DNA were combined, 26 of the total 31 detected copepod genera were represented (Fig. 5). When combining all the 18S rRNA and COI data (T‐DNA, eDNA, and PF‐eDNA), only five copepod genera identified in the morphological analysis were not detected, namely *Copilia*, *Microsetella*, *Labidocera*, *Temoropia*, and *Farranula* (Fig. 6). Even if *Microsetella* were represented in the sequence data, they would have been impossible to identify due to the lack of a reference sequence in the NCBI database; however, all the other genera are present in the database.

The genus *Acartia* was found in the PF‐eDNA and T‐DNA, but was not identified based on morphology. These samples were reanalyzed under the microscope to verify the absence of *Acartia*. None were found even after an extremely thorough assessment. Some sequences from the eDNA and T‐DNA were identified down to species (e.g. *Corycaeus quasimodo* and *Oithona simplex*). While it was challenging to definitively identify these copepods to species by microscopy, the relative abundances of these two genera were comparable between genetic and morphological approaches (Supporting Information Fig. S3). Other copepod genera had different relative abundances between the two genetic loci and sample types. The relative abundance of *Oncaea* in the 18S rRNA from T‐DNA was more comparable to the morphological assessments for all net tow mesh sizes (Supporting Information Fig. S3). Most Maxillopoda detected through 18S rRNA sequencing from the eDNA and T‐DNA samples belonged to the Calanidae family, in contrast to the morphological data, which showed dominance of the Oncaeidae family (Genus *Oncaea*). The genera *Nannocalanus* and *Clausocalanu*s were more abundant among the sequences than in the morphological assessments, with the enrichment in COI sequences more pronounced than in the 18S rRNA gene sequences.

## Discussion

### Molecular taxonomy

Metabarcoding with next‐generation sequencing is well suited for large‐scale biodiversity analyses (Shokralla et al. 2012). This technique has been successfully used to describe the diversity of mixed zooplankton tissue samples for the 18S rRNA, COI, and 28S rRNA genetic loci (Lindeque et al. 2013; Harvey et al. 2017). Our results show similar richness estimates for 18S rRNA and COI sequencing data from similarly treated samples (i.e., PF‐eDNA, eDNA, and T‐DNA) (Fig. 2).

Significantly higher total OTU richness was recovered from eDNA than from the T‐DNA. This is likely due to the diversity of organisms in the ambient waters not captured by net tows. While T‐DNA will only capture plankton within the path of the net tow, the eDNA samples will capture DNA from both benthic and pelagic (sessile and motile) animals, thus increasing the diversity in those samples.

To avoid biasing our sequence outcome by catching whole animals (i.e., copepods or nauplii) on the 0.2 μm filters used for collecting eDNA, one treatment (PF‐eDNA) involved pre‐filtering the eDNA samples through a 3 *μ*m filter. The 3 *μ*m pre‐filter was chosen based on standards in microbial and chemical oceanography, where a 3 *μ*m filter is frequently used to separate particle‐associated vs. free‐living microorganisms (Michaud et al. 2006). This prefiltration step resulted in reduced sequence recovery, reduced richness, and a bias in estimated taxonomic composition (Fig. 4c). Since eDNA is estimated to range in size between 1 *μ*m to 10 *μ*m (Turner et al. 2014), it is likely that the 3 *μ*m filter retained relevant genetic material. We, therefore, recommend against pre‐filtering; however, future studies could evaluate the effect of pre‐filtering with a pore size greater than 10 *μ*m to ensure removal of animal specimens with minimal eDNA removal.

The three molecular treatments (i.e., PF‐eDNA, eDNA, and T‐DNA) yielded significantly different taxonomic compositions, with triplicates grouping closely together with each other on the NMDS (Fig. 3). Although the triplicates for each treatment were similar, we did encounter examples where taxa were only detected in one of the triplicates. Thus, triplicate samples allowed detection of more taxa and gave higher confidence in the results due to increased statistical power in the analyses.

For the COI eDNA sequences annotated to genera (Fig. 1; Table 1), the total richness was significantly lower than for 18S rRNA eDNA sequences. This raised the question of database choice for sequence comparisons of different genetic loci. The SILVA database (Pruesse et al. 2007) is superior for annotating 18S rRNA sequences to finer taxonomic levels than the NCBI nt database (Lindeque et al. 2013). However, since COI sequences are not included in the SILVA database, this study annotated all sequences using the NCBI database to allow comparison of diversity and taxonomy between the two loci. The NCBI nt database consists of an annotated collection of all publicly available DNA sequences and is populated by both full and partial DNA sequences (Benson et al. 2012). The difference between richness of 18S rRNA and COI sequences annotated to genera from eDNA is most likely due to fewer reference sequences available for COI than for 18S rRNA. It is also possible that the intraspecific variability within this portion of the COI gene is relatively high. This would result in a higher OTU richness than that seen amongst the annotated sequences as multiple OTUs may cluster together into a single genus, such as has been shown previously for Diptera (Meier et al. 2006).

Although the NCBI nt database offers the convenience of comparing multiple loci against an identical database, better taxonomic assignments will likely be achieved through comparisons to specialized databases such as SILVA for 18S rRNA and BOLD for COI sequences (Min and Hickey 2007; Lindeque et al. 2013). Regardless of the specific database used, the taxonomic assignment of OTUs may be biased or hindered by a lack of reference sequences. As was the case for the copepod genus *Microsetella* in this study, it will be impossible to identify some taxa observed by microscopy if their sequences are not present in the databases. Additionally, submission of sequences to some databases, including NCBI, does not require voucher specimens to prove species identification, which could lead to incorrect or ambiguous annotations. We caution that until the databases are better populated, care must be taken with interpretation of sequence comparison results, especially with respect to rare or unexpected species.

### Morphological taxonomy

The most common holoplankton in Florida Bay are the copepod genera *Paracalanus*, *Oithona*, and *Acartia* (Kelble et al. 2010). However, in March 2016, samples, *Acartia* were not observed in the morphological assessments performed by microscopy. Both genetic markers detected *Acartia* in both eDNA and T‐DNA samples, however never in significant abundances. A few copepods from the genus *Acartia* were found by microscopy in the same locations during other seasons, but never more than 10 individuals. This genus may be rare and very patchy, or more actively avoids nets than other copepods (Kaartvedt et al. 2012). Database limitations and misidentification of sequences submitted to the database could also yield false positives from the sequencing data and lead to a lower or higher sequence abundance of any copepod genus. The sequencing methods can identify all life‐stages of organisms while microscopy can generally only identify adults; therefore, the detection of sequences from early life stages (eggs or nauplii) could explain the detection of copepod sequences in the absence of identifiable adults under the microscope.

### Comparison of molecular and morphological taxonomy

Morphological and molecular techniques both detected a large variety of taxa (Supporting Information Fig. S1). The eDNA samples detected a larger diversity of organisms than the T‐DNA or microscopy. Biodiversity observations from eDNA are not biased by sampling method (e.g., different mesh sizes, net avoidance, or destruction of gelatinous animals). Yet these eDNA methods are sensitive to other biases, such as the integrity of DNA exposed to temperature fluctuations, bacterial activity, variation in DNA residence time, UV exposure, or differences in DNA shedding rates by different organisms. T‐DNA performed better than eDNA at detecting the copepod genera identified in microscopy analyses. Thus, T‐DNA reflected more closely what was detected by microscopy of the net samples, likely because the T‐DNA and microscopy methods share the same net‐biases and originate from the same pool of animals. In contrast, eDNA metabarcoding was superior for detecting organisms other than copepods, while still recovering most of the dominant copepod taxa (Fig. 6) and. Thus, eDNA metabarcoding provides different insights into biodiversity of the zooplankton. Therefore, while T‐DNA is more likely to correspond slightly better with traditional morphological analyses, this study suggests that eDNA is a suitable technique for assessing the overall diversity of zooplankton communities. In addition, eDNA data from 18S rRNA seems to reflect a more similar relative abundance of taxa similar to that of morphological analysis.

We propose that differences in total abundance from morphological vs. metabarcoding assessments are due to differences in the total biomass sampled by each technique. Morphological analyses measure numerical abundance of organisms including their life‐stages, whereas metabarcoding analysis is more closely related to biomass and does not yield any information on life‐stages. The relationship between biomass and number of sequences is not linear since sequence generation is subject to biases that can be introduced at a number of different steps during sample processing such as DNA extraction, PCR, and bioinformatic classification (Bik et al. 2012). In addition, a high gene copy number for different taxa or markers could inflate the sequence abundance relative to biomass (Klappenbach et al. 2000).

The most abundant copepod genera identified by microscopy were all detected by eDNA and T‐DNA (with the exception of *Euterpina* and *Labidocera*; Fig. 6). This shows that both eDNA and T‐DNA metabarcoding will detect the dominant copepod genera of the community, with eDNA being the easiest and least invasive sample collection/analysis method with the fewest opportunities for human error. In terms of the relative abundance of copepods maxillopoda, the T‐DNA sequences reflect the morphological assessment slightly better than eDNA. Globally, most copepod communities are dominated by a few genera (e.g., *Calanus, Acartia, Oithona, Clausocalanus, Paracalanus*, and *Pseudocalanus*), which were all detected in this study, making eDNA an applicable method for analysis of dominant copepod communities from other locations as well as for the Florida Keys. The two genetic loci analyzed in this study complement each other, resulting in the identification of different taxa. Since each primer set has different associated biases, performing metabarcoding of multiple genetic loci will maximize the recovery of ecosystem biodiversity. Additional markers (e.g., 28S rRNA gene as seen in Harvey et al. (2017)) would most likely yield additional taxa, which could increase the diversity of recovered sequences.

A few copepod groups (*Euterpina*, *Pontella*, Sapphirinidae, and *Temora*) were initially detected only through sequencing and not observed by morphology. Further, targeted searches enabled their subsequent morphological identification through re‐analysis of total, as opposed to split samples (Figs. 4, 5). Subsampling (using the Folsom splitter) of the zooplankton for morphological analysis results in removal of some of the more rare taxa. This may not be the case with sequencing as some trace genetic material of all organisms will likely be present in all sub‐samples, with recovery more dependent on sequencing depth. Combining techniques (sequencing of T‐DNA and microscopy) resulted in identification of the largest number of copepod genera and would be recommended for a thorough assessment of copepod diversity in marine systems.

Each of the different sampling methods and identification techniques yielded somewhat different taxonomic composition of the zooplankton communities. Using eDNA for the detection of dominant taxa of zooplankton communities in marine systems is promising, especially when combining two or more different genetic loci. Therefore, each locus or method used represents a different “window” through which to view biodiversity. Each method complements the others, and using several different methods could yield a more complete picture of the biosphere. Our results indicate that sequencing the tissue from net tows yields the highest diversity of copepods. Although this method can be affected by limitations of databases and net‐avoidance by organisms, it reduces the time and human error of microscope counts. Diversity analyses performed using eDNA have the advantage of easier and less invasive sample collection, plus the ability to recover species not captured through net tows. Therefore, eDNA metabarcoding provides complementary insights into zooplankton biodiversity. Metabarcoding of either tissues or eDNA will become progressively more accurate for estimating diversity as genetic databases become more taxonomically enriched over time.

## Author Contribution Statement

AD and MB conceived the study. AD, NAS, JRM, BM, and EM conducted sample collection and lab analyses. Data analysis was done by AD and KP. AD drafted the manuscript. All authors revised and edited the manuscript.

## Conflict of Interest

None declared.

## Supporting information

Supporting Information
