# Supplementary material for: Evaluation of marine zooplankton community structure through environmental DNA metabarcoding
Source: Limnol Oceanogr Methods. 2018 Jan 17;16(4):209–21. doi: 10.1002/lom3.10237 (PMC5993268; doi:10.1002/lom3.10237)
Supplement: Supplementary file 1 — Supporting Information [file LOM3-16-209-s001.docx]

Supplemental figures


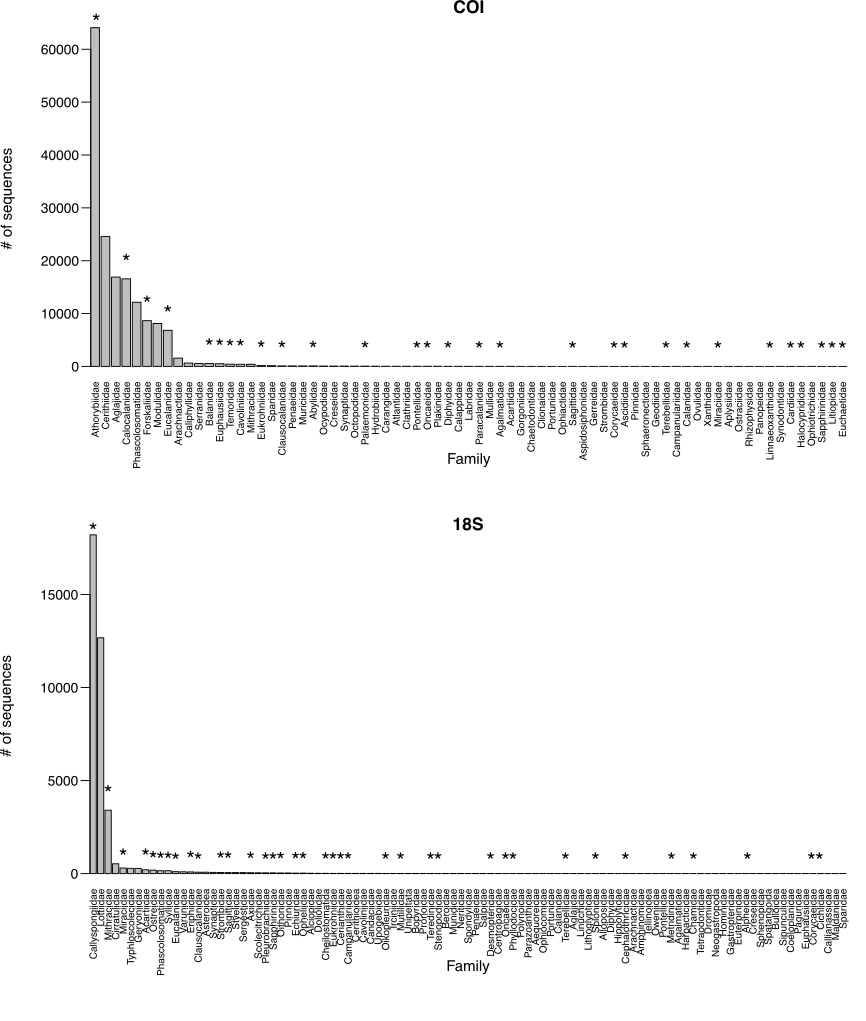


Figure S1: Rank abundance plot of all detected families from sequencing of both loci (18S rRNA and COI genes). The stars represent families detected by microscopy.

Figure S2: Scatter plot of sequence abundance and calculated biomass for the copepod genera *Oithona* and  *Paracalanus,* the copepod orders Harpacticoida and Gastropoda. Biomass conversion factors were used as in Kelble et al. 2010. The y-axes are log scaled.


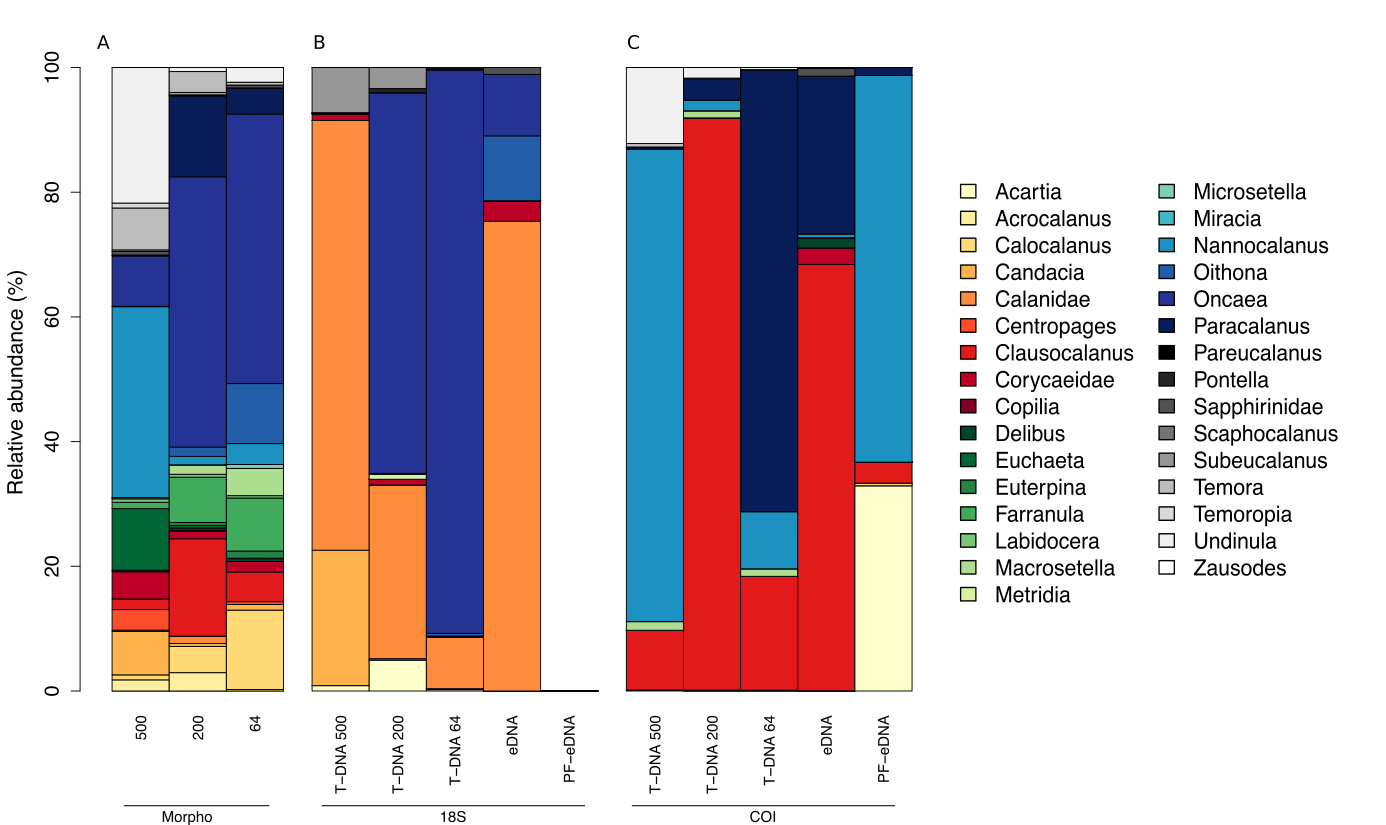


Figure S3: Barplot of all copepod data. A: morphological, B: 18S rRNA, C: COI. COI and 18S rRNA detect different relative abundances of copepods on a genus level (i.e. *Candacia*, *Clausocalanus, Nannocalanus,* and *Paracalanus*). The tissue DNA (T-DNA) resembles the zooplankton community identified by microscopy significantly better than the environmental DNA (eDNA) or pre-filtered environmental DNA (PF-eDNA), especially for 18S rRNA. PF-eDNA did not detect any copepod sequences for 18S rRNA. The numbers on the labels for each bar refer to the mesh size used in the net tows and the letters are the sampling stations (Molasses reef (MR), Looe Key (LK), and Western Sambo (WS)).
